# Netrin-1 mitigates acute lung injury by preventing the activation of the Toll-like receptor 4/nuclear factor-κB (TLR4/NF-κB) signaling

**DOI:** 10.18632/aging.205527

**Published:** 2024-02-09

**Authors:** Jian Su, Zhu Jian, Miao Zou, Huasheng Tong, Peng Wan

**Affiliations:** 1 Department of Pulmonary and Critical Medicine, The First Clinical Medical College of Three Gorges University, Yichang Central People’s Hospital, Yi Chang, Hubei 443000, China; 2 Department of Critical Care Medicine, The First Clinical Medical College of Three Gorges University, Yichang Central People’s Hospital, Yi Chang, Hubei 443000, China; 3 Department of Intensive Care Unit, General Hospital of Southern Theatre Command of PLA, Guangzhou, Guangdong 510000, China

**Keywords:** acute lung injury, lipopolysaccharide (LPS), inflammation, oxidative stress, nuclear factor-κB (NF-κB), Netrin-1 (NT-1)

## Abstract

Acute lung injury (ALI) is one of the most common high-risk diseases associated with a high mortality rate and is still a challenge to treat effectively. Netrin-1 (NT-1) is a novel peptide with a wide range of biological functions, however, its effects on ALI have not been reported before. In this study, an ALI model was constructed using lipopolysaccharide (LPS) and treated with NT-1. Pulmonary function and lung wet to dry weight ratio (W/D) were detected. The expressions of pro-inflammatory cytokines and chemokines interleukin-8 (IL-8), interleukin-1β (IL-1β), and chemokine (C-X-C motif) ligand 2 (CXCL2) were measured using real-time polymerase chain reaction (RT-PCR) and enzyme-linked immunosorbent assay (ELISA). We found that the levels of NT-1 were reduced in the LPS-induced ALI mice model. Administration of NT-1 improved histopathological changes of lung tissues and lung function in LPS-challenged ALI mice. We also report that NT-1 decreased Myeloperoxidase (MPO) activity and ameliorated pulmonary edema. Additionally, treatment with NT-1 reduced the levels of pro-inflammatory cytokines and chemokines such as IL-8, IL-1β, and CXCL2 in lung tissues of LPS-challenged ALI mice. Importantly, NT-1 reduced cell count in BALF and mitigated oxidative stress (OS) by reducing the levels of MDA and increasing the levels of GSH. Mechanistically, it is shown that NT-1 reduced the levels of Toll-like receptor 4 (TLR4) and prevented nuclear translocation of nuclear factor-κB (NF-κB) p65. Our findings indicate that NT-1 is a promising agent for the treatment of ALI through inhibiting TLR4/NF-κB signaling.

## INTRODUCTION

Acute lung injury (ALI) is a clinical syndrome induced by damages to lung tissue, leading to various pathological and structural changes, characterized by alveolar injury, pulmonary edema development, neutrophil-produced inflammation, and surfactant malfunction [1, 2]. Clinically, ALI is manifested as decreased pulmonary compliance, severe hypoxemia, and bilateral pulmonary infiltrates. The American-European consensus criteria (AECC) defines ALI as the presence of acute onset, bilateral pulmonary infiltrates on chest radiography, pulmonary arterial wedge pressure ≤18 mmHg or no left atrial hypertension, and PaO2/FiO2 ≤300 mmHg if present [3, 4]. ALI has a high incidence, with over three million cases diagnosed worldwide each year, accounting for 10% of Intensive Care Units (ICU) admissions [5]. The mortality rate associated with the condition is even more concerning than the incidence rate. In a study conducted in King County, Washington, the incidence of ALI in children aged 0.5–15 years was 12.8 cases per 10000 people per year, with a mortality rate of 18% [6]. Unbridled pulmonary inflammation is the primary mechanism underlying ALI [7]. In the inflammatory process of ALI, cytokines such as IL-8, IL-1β, and CXCL2 mediate the accumulation and infiltration of various immune cells into lungs, activating intracellular signaling pathways and releasing a large number of cytokines. Immune cells are continuously activated to form a vicious cycle, ultimately leading to cytokine storms [8]. OS also closely participates in ALI processing. When exposed to ALI risk factors, excessive reactive oxygen species (ROS) are generated. Cells typically express several enzymes to safeguard from OS damage [9], however, excessive ROS that exceed the antioxidant capacity cause lipid peroxidation in cell membranes, leading to pulmonary edema and pulmonary expansion [10]. Thus, regulating inflammatory reactions and OS may become important research directions for treating ALI. LPS is a major component of the outer membranes of gram-negative bacteria, and increasing research indicates that gram-negative bacterial infection is one of the most important causes of ALI. LPS is able to cause lung injury and elicit inflammatory response [11, 12], thus, LPS-induced ALI in mice has become a well-accepted model for disease investigation [13]. NT-1 is a biologically active molecule that has multiple critical biological functions, it closely participates in the development and function of the nervous system and is therefore called a neural guidance molecule [14, 15]. NT-1 exerts a vital function in the growth and guidance of axons during embryonic development, especially in the central nervous system, which affects the establishment of neuronal networks and the formation of synapses, helping to build the complexity of neuronal networks. In the absence of NT-1, axons may fail to orient correctly, leading to defects in neuronal network formation [16]. NT-1 is recently claimed to alleviate cirrhosis by inhibiting the inflammatory response mediated by the UNC5b/Peroxisome proliferator-activated receptor γ (PPARγ) signaling axis [17]. In addition, NT-1 alleviates cerebral reperfusion injury by blocking OS by limiting mitochondrial ROS release [18]. However, the therapeutic potential of NT-1 for ALI remains unknown. In this study, we aim to investigate the potential beneficial effects of NT-1 in ALI using an animal model. Also, we examine the underlying mechanism whereby NT-1 exerted its protective actions by examining oxidative stress and inflammatory response. The involvement of TLR4/NF-κB signaling has also been assessed.

## MATERIALS AND METHODS

### ALI modeling

Forty-eight male C57BL/6 mice aged between 6 to 8 weeks old were used. Each mouse was anesthetized with 45 mg/kg pentobarbital sodium in 100 μL, fixed in the supine position, and then 50 μg LPS solution was dropped into the nostril, dissolved in 20 μL physiological saline. Before dropping the LPS solution, the mouse’s tongue was gently pulled out using ophthalmic forceps to prevent swallowing of the LPS liquid. At the same time, the mouse’s respiratory movement was observed, and the LPS solution was dropped into the mouse’s nose when it took a deep breath. The LPS solution was dropped slowly and repeatedly. Finally, the mouse was gently rotated to promote uniform distribution of LPS in the lungs [19].

### Grouping

The mice were divided into 4 groups: Vehicle, NT-1, LPS, and LPS+NT-1. In the NT-1 and LPS+NT-1 groups, normal mice and LPS mice were intraperitoneally injected with 80 μg/mL mouse Netrin-1 protein (R&D, 1109-N1-025, USA) for 1 week before the ALI model induction. In the vehicle and LPS groups, normal mice and LPS mice were injected with the same volume of normal saline [20].

### Enzyme-linked immunosorbent assay (ELISA)

The following kits were used in this experiment: mice NT-1 ELISA kit (JINMEI BIOTECHNOLOGY, Yancheng, China), mice IL-8 ELISA kit (Cat#FT-P9S3048X, Shanghai Fan Tai Biotechnology, Shanghai, China), mice IL-1β ELISA Kit (Cat#E-EL-M0037c, Elabscience, Wuhan, China), and mice CXCL2 ELISA Kit (Cat#ml058180, Shanghai Enzyme-linked Biotechnology Co., Ltd, China). MPO activity in lung tissues was determined using the ELISA assay (Cat#EEA016, Invitrogen, USA). The microplate, which had already been coated with the antibody, was successively added with serum samples, standards, and horseradish peroxidase (HRP). Afterwards, the reaction plate was placed in a constant temperature water bath to allow for a full reaction. After thoroughly washing the plate, the substrate 3.3′5.5′ tetramethylbenzidine (TMB) was added for color development, and under the action of peroxidase and acid, the substrate color changed from blue to yellow. Finally, the wavelength of the reader was set to 450 nm, and the optical density (OD) values of each microplate were measured. Based on the standard curve equation, the concentrations of each sample were further calculated.

### Real-time polymerase chain reaction (RT-PCR) assay

Total RNA was extracted from the lung tissue using TRIzol (Cat#15596026, Invitrogen, USA) and quantified using NanoDrop Lite (Thermo Fisher Scientific, USA). 2 μg total RNA was reverse transcribed into cDNA using a cDNA kit (Takara, Japan). The SYBR Green assay kit (Takara, Japan) and real-time PCR instrument (Bio-Rad, USA) were used for real-time PCR. Expression levels of mRNA were normalized using the 2^−ΔΔCT^ method, and the mRNA expression levels of the relevant genes, IL-1β, IL-8, and CXCL2, were compared with the internal reference gene Glyceraldehyde-3-phosphate dehydrogenase (GAPDH) [21].

### The detection of pulmonary function

Mice were anesthetized utilizing pentobarbital sodium (90 mg/kg) and their spontaneous breathing was completely suppressed. The trachea was exposed and connected to a pulmonary function analyzer (DSI Buxco, USA), with a frequency setting of 90, a respiratory ratio of 15:10, and a tidal volume of 5 mL/kg. The body contour was monitored using a computer, and relevant pulmonary function indicators were recorded, including resistance of airway (RAW), peak expiratory flow (PEF), and dynamic lung compliance (Cdyn), etc. The mice were then removed after 30 min.

### Lung wet to dry weight ratio (W/D) detection

After mice were sacrificed, lungs were carefully and completely dissected using ophthalmic scissors. The right lung without lavage was taken and the surface liquid was absorbed using filter paper. The weight was measured and recorded (wet weight). The temperature was set at 60°C and the drying time was set to more than 48 h. Then, the weight was measured again (dry weight). The formula R = W/D was used to calculate the ratio [22].

### Cell counting in bronchoalveolar lavage fluid (BALF)

A pair of scissors with a suitable angle was chosen to make a small horizontal incision on the trachea just below the animal’s throat. A 1-milliliter syringe filled with buffered salt solution (HBSS) was gently inserted into the cannula to inject 900 microliters slowly and continuously into the trachea, inflating the lungs. The BALF was removed immediately, followed by centrifugation to discard the supernatant. The cell precipitate was collected, and 1 milliliter of PBS solution was added. A cell counter (Beckman, USA) was used to determine counts of leukocytes and neutrophils [23].

### The detection of the protein concentrations in BALF

The BALF of each animal was collected to check the concentrations of protein utilizing the bicinchoninic acid (BCA) method (Keygen, China), with the instructions on the kit strictly followed.

### The detection of MDA and GSH content in the lung tissue

The 0.02% 2-thiobarbituric acid reagent was weighed out, buffered with buffer solution to a concentration of 2 mg/ml TBA solution, then wrapped in tin foil and stored in a refrigerator at 4°C. The lung tissues were collected, weighed and recorded, followed by being cut into small pieces, mixed with a small amount of physiological saline solution, and made into a 10% tissue homogenate. The supernatant was discarded following centrifugation to collect the precipitate. 5% trichloroacetic acid was added to the precipitate, mixed evenly, and centrifuged at 10000 r/min for 10 min. The supernatant was collected and 0.4 volume of chloroform-isopentanol mixture (volume ratio of 2:1) was added to the supernatant, mixed gently with a glass stick, centrifuged at 10000 r/min for 10 minutes, and the supernatant was collected. The MDA content was calculated by measuring the absorbance at a wavelength of 532nm and a light path length of 1 cm, while the reduced GSH content was calculated by detecting the absorbance at a wavelength of 412 nm and a light path length of 1 cm, using a visible spectrophotometer (Shimadzu, Japan).

### Western blotting assay

100 mg of mouse lung tissue was chopped and incubated in a mixture of strong RIPA lysis buffer and PMSF (100:1) to extract cytoplasmic and nuclear proteins. The protein content of the lung tissue was determined with a BCA protein quantification kit (Keygen, China). The volumes of 5× loading buffer and diluent were calculated and mixed with the lung tissue protein solution, followed by boiling, cooling, and aliquoting. Separation and concentration gels were prepared, samples were loaded, electrophoresis was performed, membranes were transferred, and the membranes were blocked. Primary antibodies (TLR4, 1:1000; NF-κB p65, 1:800; β-actin, 1:1000, CST, USA) were incubated overnight, washed with TBST, and incubated with secondary antibodies (1:6000, CST, USA) for 2 h. Following washing, blots were exposed. Finally, the ImageJ software was applied for analysis [24].

### Statistical analysis

All data were presented as “mean ± standard deviation”, and all bar graphs were generated using GraphPad Prism 8. The statistical significance of all data was analyzed using one-way ANOVA and Tukey’s Post hoc test. *P* < 0.05 indicated significant differences, and *P* < 0.01 indicated extremely significant differences.

### Data availability statement/availability of data materials

The data that support the findings of this study are available from the corresponding author upon reasonable request.

## RESULTS

### The levels of serum NT-1 were reduced in ALI mice

**Figure 1:**
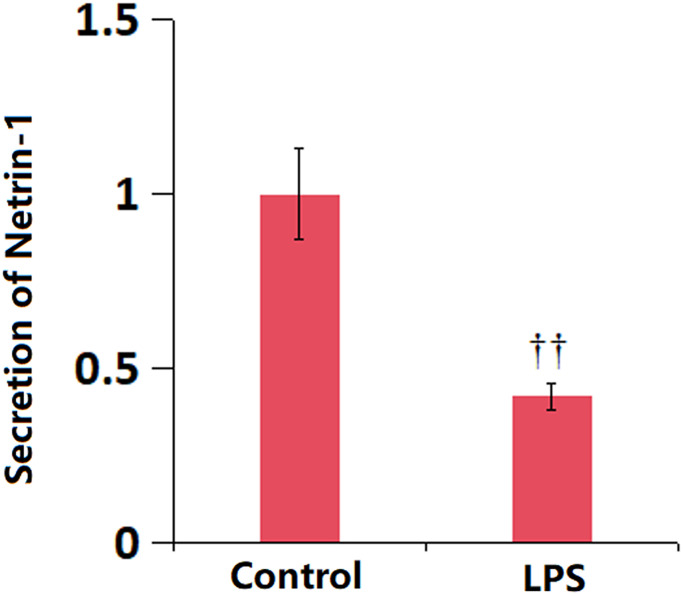
**The levels of serum Netrin-1 were reduced in LPS-induced ALI mice model.** The levels of serum Netrin-1 were measured using ELISA (^††^*P* < 0.01 vs. vehicle group).

To predict the possible function of NT-1 in ALI, the serum NT-1 level in ALI mice was detected. The serum level of NT-1 was remarkably declined in ALI mice (Figure 1), implying a protective function of NT-1 in ALI.

### NT-1 improved histopathological changes and lung function in ALI mice

**Figure 2:**
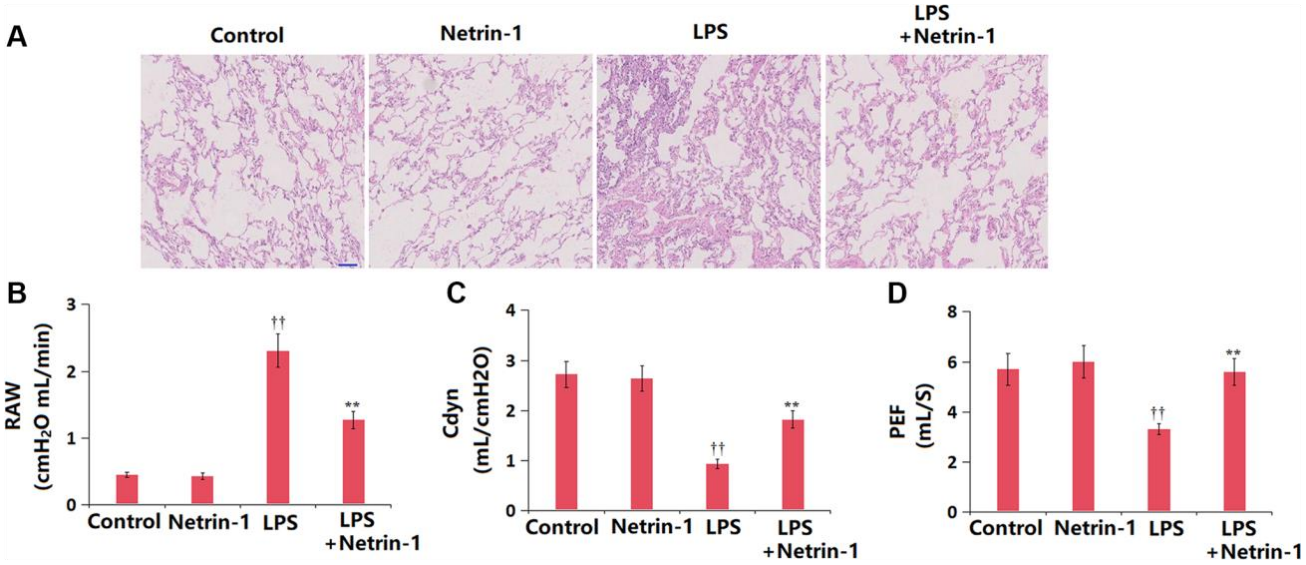
**Netrin-1 improved histopathological changes and lung function in lung tissues in LPS- challenged ALI mice.** (**A**). Histopathological changes of the lung tissue; Scale bar, 100 μm; (**B**). RAW (cmH_2_O mL/min); (**C**). Cdyn (mL/cmH2O); (**D**). PEF (mL/S) (^††^*P* < 0.01 vs. vehicle group; ^**^P < 0.01 vs. LPS group).

In the vehicle and NT-1 groups, there were no manifestations of ALI, and the color was uniform and pink. In the ALI group, the lung tissue was extensively swollen and showed diffuse ecchymoses, with obvious pulmonary consolidation and foam-like secretions exuding from the trachea. These histopathological changes observed in ALI mice were sharply alleviated by NT-1 (Figure 2A). The values of RAW (Figure 2B) in the control, NT-1, ALI, and NT-1+ALI groups were 0.45, 0.43, 2.31, and 1.27 cmH_2_O mL/min, respectively. Moreover, the Cdyn value was slightly altered from 2.72 to 2.64 mL/cmH_2_O in the NT-1 group, markedly declined to 0.93 mL/cmH_2_O in ALI mice, then largely elevated to 1.82 mL/cmH_2_O by NT-1 (Figure 2C). The PEF values in the control, NT-1, ALI, and NT-1+ALI groups were 5.7, 6.0, 3.3, and 5.6 mL/S, respectively (Figure 2D).

### NT-1 decreased MPO activity and edema in ALI mice

**Figure 3:**
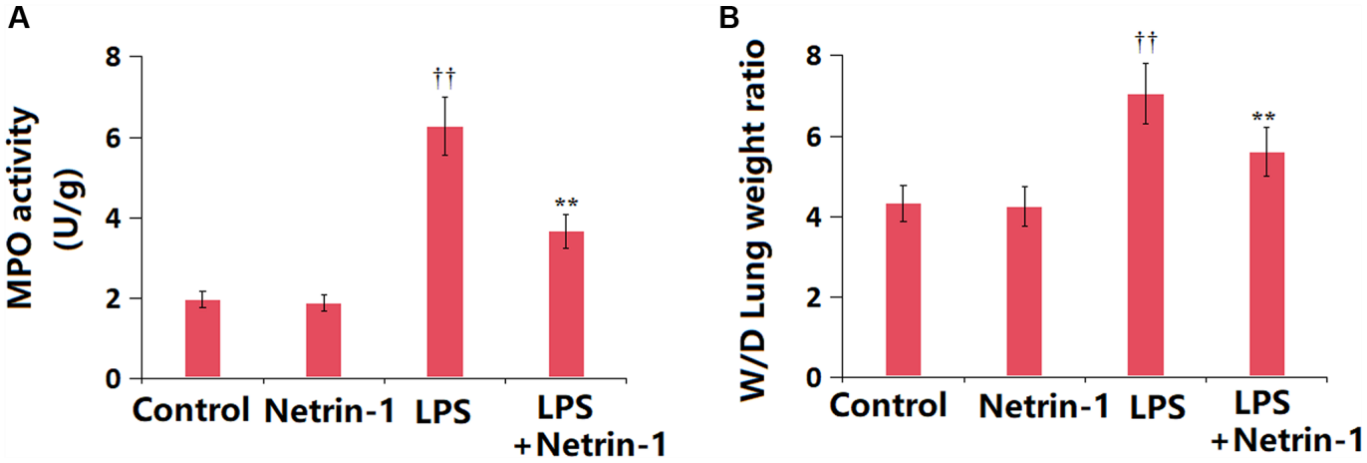
**Netrin-1 decreased MPO activity and pulmonary edema in LPS-challenged ALI mice.** (**A**) MPO activity; (**B**) Lung wet to dry weight ratio (^††^*P* < 0.01 vs. vehicle group; ^**^P < 0.01 vs. LPS group).

The activity of MPO was changed from 1.96 to 1.87 U/g in the NT-1 group, was notably increased to 6.27 U/g in ALI mice, then remarkably reduced to 3.65 U/g by NT-1 (Figure 3A). Furthermore, the lung W/D ratio in the control, NT-1, ALI, and NT-1+ALI groups was 4.31, 4.25, 7.05, and 5.61, respectively (Figure 3B).

### NT-1 inhibited inflammation in ALI mice

**Figure 4:**
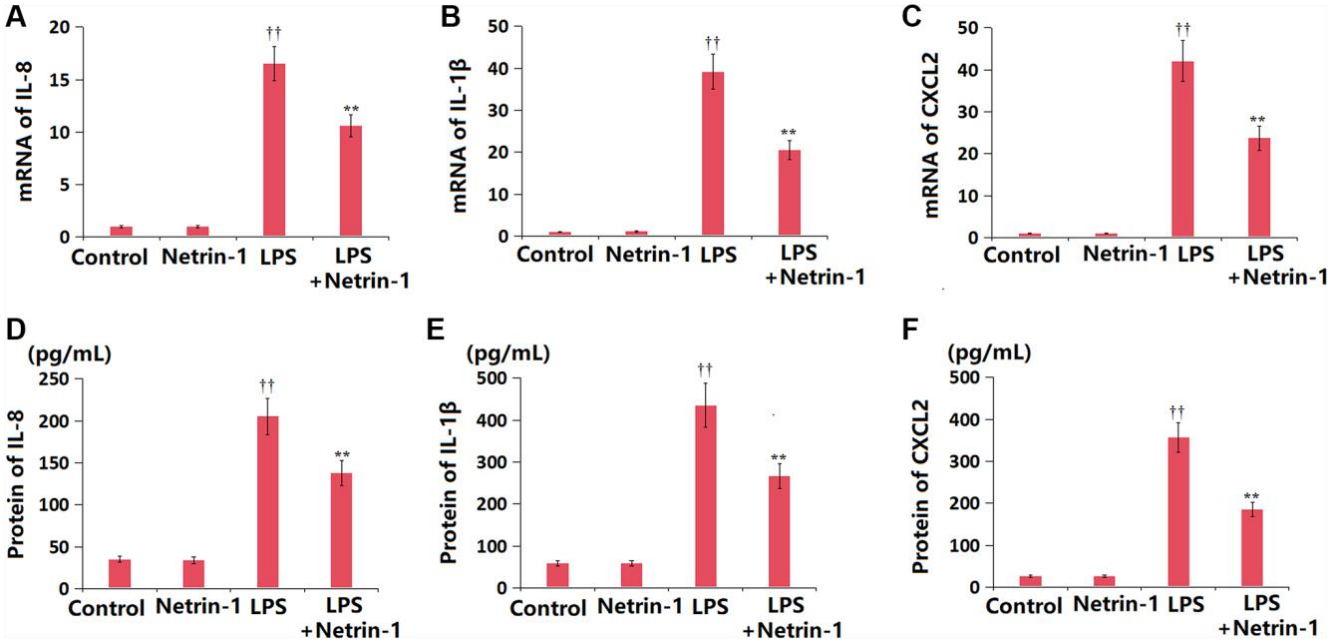
**Netrin-1 inhibited the expression of pro-inflammatory cytokines and chemokines in lung tissues in LPS-challenged ALI mice.** (**A**) mRNA of IL-8; (**B**) mRNA of IL-1β; (**C**) mRNA of CXCL2; (**D**) Protein of IL-8; (**E**) Protein of IL-1β; (**F**) Protein of CXCL2 (^††^*P* < 0.01 vs. vehicle group; ^**^P < 0.01 vs. LPS group).

Slightly altered levels of IL-1β, IL-8, and CXCL2 were observed in the NT-1 group, all of which were largely increased in ALI mice, then sharply repressed by NT-1 treatment (Figure 4A–4C). The IL-8 content was altered from 35.1 to 33.8 pg/mL in the NT-1 group, was notably increased to 205.5 pg/mL in ALI mice, then remarkably repressed to 137.4 pg/mL by NT-1 (Figure 4D). The content of IL-1β in the control, NT-1, ALI, and NT-1+ALI groups was 58.2, 57.9, 435.6, and 267.1 pg/mL, respectively (Figure 4E). Moreover, the CXCL2 level was changed from 25.2 to 26.3 pg/mL in the NT-1 group, was signally increased to 356.5 pg/mL in ALI mice, then largely suppressed to 185.2 pg/mL by NT-1 (Figure 4F).

### NT-1 reduced cell counts in BALF of ALI mice

**Figure 5:**
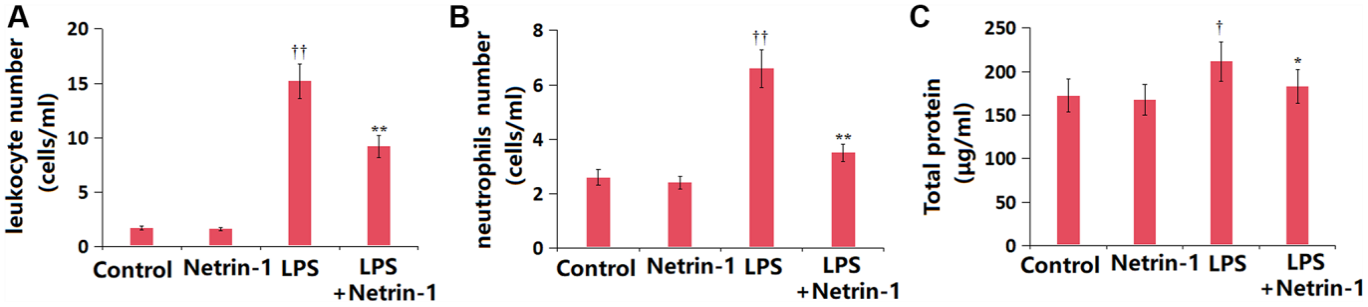
**Netrin-1 reduced cell count in BALF of LPS-challenged ALI mice.** (**A**) Total leukocyte number in BALF; (**B**) Total neutrophils in BALF; (**C**) Total protein concentrations in BALF (^†^, ^††^*P* < 0.05, 0.01 vs. vehicle group; ^*^, ^**^P < 0.05, 0.01 vs. LPS group).

Subsequently, the counts of main lymphocytes were detected in BALF. The total leukocyte number in BALF was altered from 1.7 to 1.6 × 10^7^ cells/mL in the NT-1 group, was largely increased to 15.3 × 10^7^ cells/mL in the ALI group, then remarkably reduced to 9.2 × 10^7^ cells/mL by NT-1 (Figure 5A). The total number of neutrophils in BALF in the control, NT-1, ALI, and NT-1+ALI groups was 2.6, 2.4, 6.6, and 3.5 × 10^7^ cells/mL, respectively (Figure 5B). In addition, the total protein content of BALF in the NT-1 group was slightly changed from 172.3 to 167.5 μg/mL, was signally elevated to 211.8 μg/mL in ALI mice, then markedly decreased to 183.2 μg/mL by NT-1 (Figure 5C).

### NT-1 attenuated OS in ALI mice

**Figure 6:**
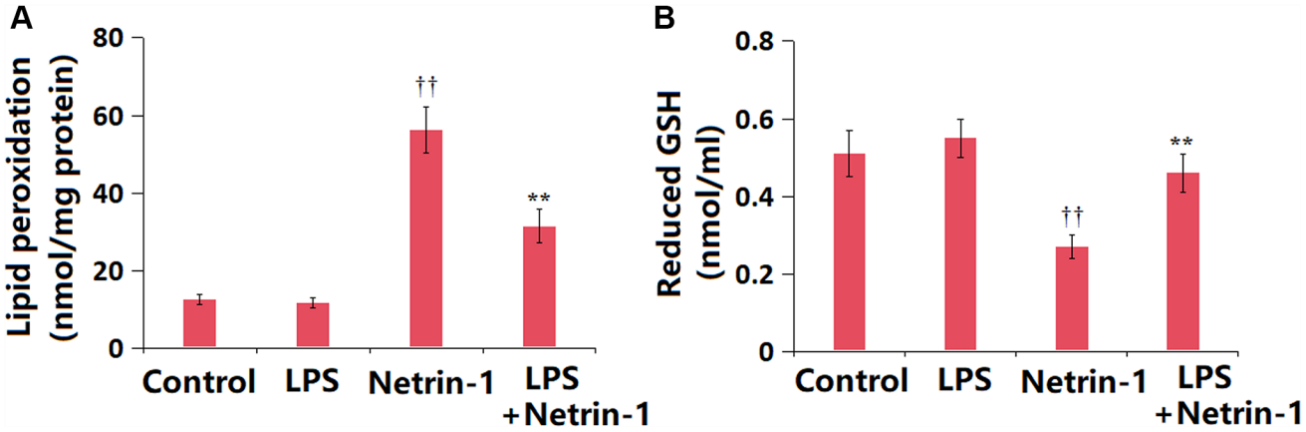
**Netrin-1 attenuated oxidative stress in LPS-challenged ALI mice.** (**A**) Lipid peroxidation was assayed by measuring MDA content; (**B**) The levels of reduced GSH (^††^*P* < 0.01 vs. vehicle group; ^**^P< 0.01 vs. LPS group).

OS is one of the main inducers of ALI [25]. The MDA content in the control, NT-1, ALI, and NT-1+ALI groups was 12.6, 11.7, 56.2, and 31.5 nmol/mg protein, respectively (Figure 6A). Moreover, the GSH content was changed from 0.51 to 0.55 nmol/mg protein in the NT-1 group, was notably declined to 0.27 nmol/mg protein in ALI mice, then significantly increased to 0.46 nmol/mg protein by NT-1 (Figure 6B).

### NT-1 inactivated the TLR4/NF-κB axis in ALI mice

**Figure 7:**
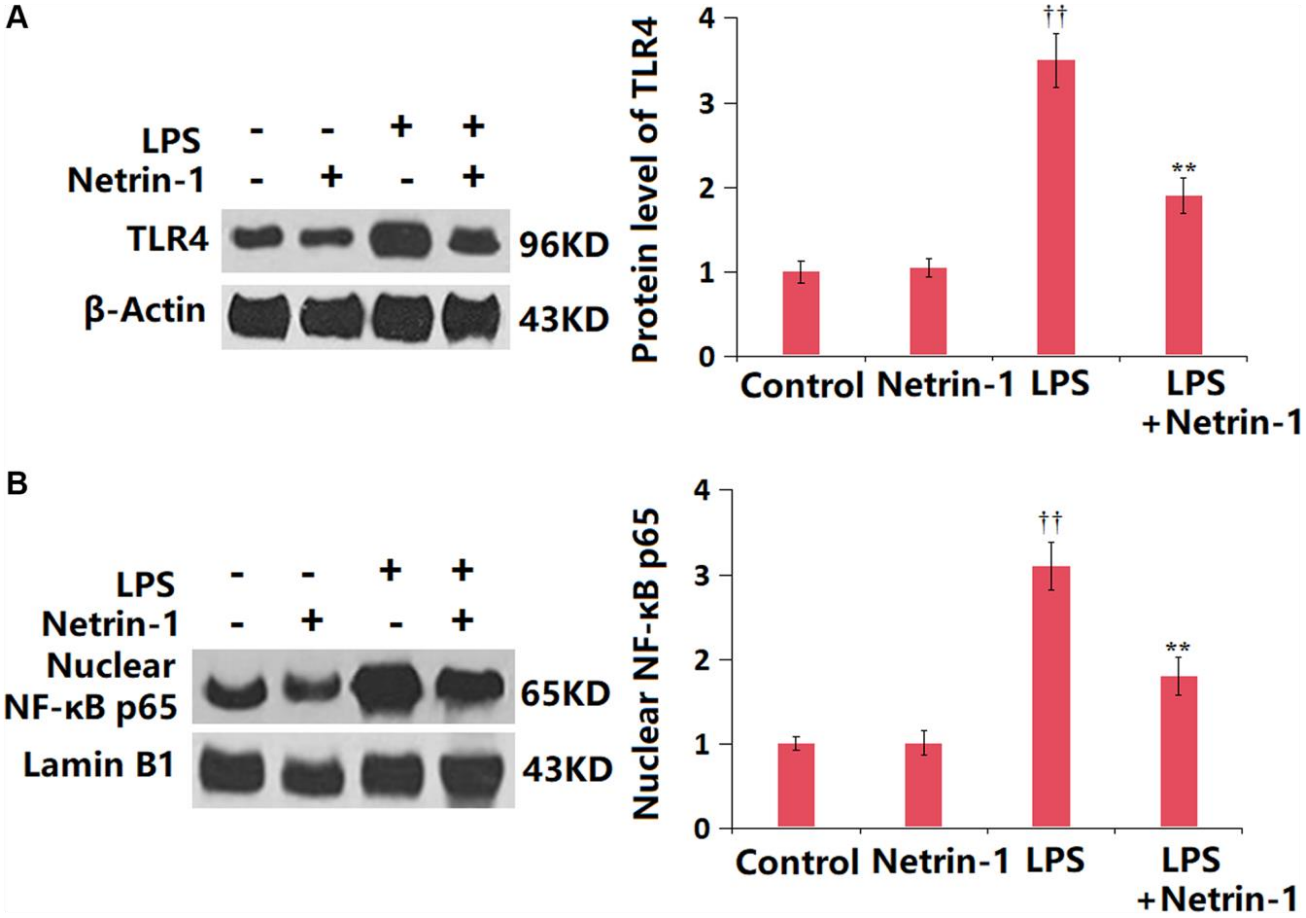
**Netrin-1 prevented activation of the TLR4/NF-κB signaling in the lung tissue in LPS- challenged ALI mice.** (**A**) The levels of TLR4; (**B**) Levels of nuclear NF-κB p65 were measured by western blot analysis (^††^*P* < 0.01 vs. vehicle group; ^**^P < 0.01 vs. LPS group).

The TLR4/NF-κB axis is partly responsible for the inflammatory response in ALI development [26]. The TLR4 and NF-κB p65 levels in the NT-1 group were slightly altered, but were dramatically increased in ALI mice, then significantly repressed by NT-1 (Figure 7A, 7B).

## DISCUSSION

The basic pathophysiological changes of ALI are due to the destruction of pulmonary endothelial and epithelial barriers resulting in pulmonary edema (non-cardiogenic). The pathological features of ALI involve the loss of pulmonary alveolar capillary membrane integrity, excessive trafficking of neutrophils across the epithelium, and the release of pro-inflammatory cytotoxic mediators [27]. The pulmonary alveoli and capillaries are lined by type I and type II alveolar epithelial cells, respectively, and the integrity of the pulmonary endothelial vascular endothelium is crucial for gas exchange and pulmonary edema formation. Neutrophils are a frequent mechanism for the breakdown of vascular integrity [28, 29]. When damage occurs, neutrophils accumulate in the pulmonary capillary system and are stimulated, leading to the release of various harmful mediators, including proteases, reactive oxygen radicals, pro-inflammatory cytokines and thrombogenic substances, causing enhanced permeability of blood vessels to generate edema [30, 31]. Neutrophils participate in innate immunity [32, 33], the depletion of which prevents the development of ALI. It is claimed that the host experiences severe endothelial damage without experiencing alveolar epithelial damage. In preclinical models, edema, a signature feature of ALI, does not occur until alveolar epithelial damage occurs [34]. Typically, alveolar epithelial cells construct tight junctions and selectively regulate the flow of liquid through the epithelial barrier. Under pathological conditions, with the massive migration of neutrophils, injured epithelial cells cause increased permeability during ALI and allow protein-rich edema fluid to be deposited in the alveolar spaces. The damage to epithelial cells also disrupts the normal means of removing edema through Na+ channels and Na+/K+ ATPase pumps [35]. Injury to type II alveolar epithelial cells also leads to a reduction in surfactant production, reducing overall lung compliance [36]. Type II pneumocytes play a key role in epithelial regeneration, and their malfunction can lead to a pathological fibrotic repair process. All these pathogenic factors contribute to pulmonary alveolar damage, which is a hallmark of ALI [1, 3]. Herein, as referred to in previous studies [37], the ALI model was constructed in mice by administering LPS. Severe histopathological changes, impaired lung function, decreased MPO activity, and serious pulmonary edema were observed in ALI mice, in line with data reported by Zou [38] and Zhu [39]. These pathological changes in lung tissues were remarkably alleviated by NT-1, revealing the anti-ALI property of NT-1.

Inflammation is an important component of innate immune reactions that protect tissues from damage. However, when the balance in the body is disrupted, inflammation causes damage to tissues and organs. The pathogenesis of ALI can also be attributed to prolonged or excessive inflammatory processes that lead to lung tissue damage [40]. Numerous studies have shown that during the sepsis induced by LPS, inflammatory cells produce large amounts of inflammatory mediators that play a crucial role in the disease progression [25]. For instance, TNF-α activates neutrophils to adhere to lung tissue and release large amounts of oxygen radicals. Additionally, in the process of ALI, a large amount of fluid accumulates in the alveoli and pulmonary interstitium, leading to pulmonary edema [41, 42]. Herein, in line with results presented by Li [43], the enhanced inflammation was observed in ALI mice, accompanied by increased infiltration of lymphocytes and protein content in BALF, which were observably ameliorated by NT-1, revealing that the anti-ALI function of NT-1 might result from its anti-inflammatory role. A previous study has shown that OS damage is an important factor in ALI, and the main degradation product of cell lipid peroxidation is MDA, which can indirectly reflect the extent of cell oxidative damage [44]. Reducing the level of MDA and activating the SOD activity will significantly alleviate the pathological changes of lung tissue caused by endotoxin [45]. Herein, OS was enhanced in lung tissues of ALI mice, which was remarkably alleviated by NT-1. During the development of LPS-induced ALI, activation of TLR4/NF-κB signaling is involved [46]. TLR4 specifically recognizes and binds to LPS, activating NF-κB and inducing inflammation, initiating endogenous immune reactions that cause tissue and organ damage [47]. Herein, the TLR4/NF-κB axis was also found to be activated in ALI mice, which was markedly repressed by NT-1, hinting that NT-1 exerted its anti-ALI function by mediating the TLR4/NF-κB axis. Moreover, in normal mice, no remarkable changes were observed following NT-1 administration, suggesting that NT-1 would not bring obvious impact under normal physiological states. In future work, the influence of NT-1 on the TLR4/NF-κB axis will be further studied in *in vitro* assays, such as LPS-stimulated macrophages or bronchial epithelial cells.

## CONCLUSION

In conclusion, NT-1 mitigated ALI in mice by preventing TLR4/NF-κB activation. Our findings provide strong support for the possibility of NT-1-mediated anti-inflammatory interventions for ALI.
